# Enhancing Public Health Education Through Smart Learning Environments: Integrating Technology and Pedagogy

**DOI:** 10.1007/s40670-025-02408-6

**Published:** 2025-05-22

**Authors:** Sean Mark Patrick, Noel Nicholas, Melissa Maritz, Jacqueline Elizabeth Wolvaardt

**Affiliations:** 1 School of Health Systems and Public Health, University of Pretoria, Pretoria, South Africa; 2 Department of Education, University of Pretoria, Pretoria, South Africa

**Keywords:** Community of inquiry, Digital learning, Online teaching, Self-regulated learning, Smart learning environments

## Abstract

The landscape of public health education is transforming, and online learning technologies are rapidly being adopted. This narrative review examines how public health education has evolved by comparing traditional and online learning environments, focusing on how smart learning environments can enhance educational outcomes. The analysis is grounded in the community of inquiry framework, Gardner’s theory on multiple intelligences, and the theory of self-regulated learning. This review first outlines the key competencies required by public health professionals, including epidemiology, health policy, and biostatistics, and then discusses how these competencies are taught traditionally. This description is followed by an exploration of online learning environments and the unique challenges faced by learners, such as the need for self-regulation and motivation. Smart learning environments should integrate adaptive learning technologies and personalized learning pathways to address these challenges and provide a more engaging and supportive learning experience. These technologies enable real-time feedback and customization of learning materials, allowing students to monitor their progress and adjust their learning strategies. Smart learning environments can also contribute to community and collaborative learning in online environments. Integrating smart learning environments into public health curricula can enhance student learning outcomes and prepare future public health professionals to navigate a rapidly changing digital world. The purpose of this review is to contribute to the ongoing discourse on the modernization of public health education by proposing a conceptual community of inquiry framework for smart learning environments, suggesting a balanced approach that leverages the strengths of traditional and online learning methods.

## Introduction

In South Africa, more public health professionals need to be trained, which is reflected in the Human Resources for Health (HRH) Strategy for South Africa [25] and the newly passed National Health Insurance Bill [26]. This national need reflects a broader global challenge. Worldwide, there is a critical shortage and maldistribution of public health professionals, particularly in low- and middle-income countries, which undermines efforts to strengthen health systems and achieve universal health coverage. Global frameworks such as the Sustainable Development Goals (SDGs) and initiatives led by the World Health Organization emphasize the importance of a well-trained public health workforce to address complex health challenges. Including data on global workforce needs highlights the urgency of investing in public health training and situates South Africa’s efforts within a shared international imperative to build resilient health systems [50]. Individuals studying public health are prepared to plan, manage, and evaluate public healthcare services and to function as public health professionals. Public health professionals should thus be trained in epidemiology, biostatistics, public health principles, preventive medicine, health policy and regulation, healthcare services and related administrative functions, public health law enforcement, health economics and budgeting, public communications, and professional standards and ethics [11]. The Association of Schools of Public Health in Africa (ASPHA) also promotes the development of students’ skills in community dynamics, cultural contexts, and ethics. These components are essential for comprehensive public health training and research, fostering competencies, building partnerships, and enabling course accreditation through peer assessments [3]. Building on these foundational competencies, public health education must also remain responsive to the evolving teaching and learning landscape, where technology, pedagogy, and learner autonomy increasingly intersect.

The teaching and learning landscape is constantly changing, and new technologies are combined with traditional teaching methods to enhance the educational experience and drive meaningful educational reform [46]. Completing an online program is associated with several factors, such as self-regulated learning, self-directed learning, and online learning self-efficacy [15]. According to Tinto [44], the perceived value of learning activities also influences student persistence and motivation.

Thus, in this paper, we argue that students who are actively engaged in online modules and who are presented with self-regulated learning opportunities have enhanced knowledge retention and application. This narrative review proposes a conceptual framework for creating a smart learning environment (SLE) that incorporates meaningful learning opportunities for online public health students in line with the expected competencies, while allowing for varied learning preferences.

## Challenges and Opportunities in the Changing Education Landscape

Several challenges have been identified in online learning environments. Online learning is a mode of asynchronous learning that allows students to learn at their own pace and fit coursework around their schedules. Online education relies heavily on technology and internet access, and students without access may struggle to engage with learning materials and activities [18, 20]. Moreover, online learning lacks the face-to-face interaction and social dynamics of traditional classrooms, which can hamper collaborative learning, peer support, and interpersonal skill development [8]. Compared with face-to-face settings, online learning environments may also have fewer opportunities for personalized support. Students who require additional guidance or individualized assistance may struggle in online courses, negatively impacting their performance [16]. Students who engage in online learning require self-discipline, self-motivation, and effective time-management skills (Santelli et al., 2020). Students who lack these skills may have difficulty managing their schedules, resulting in poor engagement and academic performance [21].

Educators regularly encounter nonperforming students who may exhibit low interaction or engagement with course materials and activities [37]. In online environments, educators may struggle to assess student learning and comprehension due to limited opportunities for direct observation and the absence of immediate feedback [24].

In contrast, traditional, synchronous learning provides immediate feedback and fosters community through real-time interactions with educators and peers [12]. Educators frequently value the opportunities for immediate clarification, discussion, and real-time assessment provided by synchronous teaching. Alternatively, asynchronous teaching allows for greater scalability, accommodates diverse student needs, and offers more time to reflect and elaborate on ideas [17]. Students and educators value developing a sense of community and social presence in online courses [33]. This presence includes facilitating introductions, icebreakers, and interactions through discussion forums, group activities, and online meetings [23]. Students may experience a greater sense of belonging when they are actively engaged in learning. Educators can encourage student participation by implementing online instructional strategies, such as problem-solving activities, peer collaboration, and student-led discussions [10, 43]. Educators must communicate effectively with students to develop this sense of belonging, fostering a supportive learning environment through regular and timely feedback, responsive communication, and addressing individual needs and concerns [29]. While some students may experience engagement, actively participate, interact with peers, and feel a sense of connection with educators and peers online [21], others experience isolation, a lack of interaction, and a poor sense of community due to differences in digital literacy, learning preferences, access to stable internet, and the design or facilitation of the online learning environment itself[34, 41].

## Smart Learning Environments and Gardner’s Theory of Multiple Intelligences

Smart learning environments (SLEs) are technology-enhanced systems that adapt to learners’ needs using real-time data, personalized feedback, and flexible instructional design. They support individualized learning and promote engagement, aligning well with diverse educational approaches and various learning styles and intelligences [22]. Howard Gardner’s theory of multiple intelligences asserts that learning opportunities, assessments, and the broader learning environment should be designed to accommodate the diverse intelligences of students. Students should be allowed to tailor the information to their learning styles, enhancing their educational experience [13]. Gardner [13] identified eight intelligences that can be mapped against the core competencies expected of public health professionals (Table 1). The application of Gardner’s multiple intelligences ensures that all core competencies are effectively addressed, fostering an adaptable and flexible learning environment. Using a flexible approach in public health education may provide a workforce capable of adapting to rapidly changing demands and emerging health threats. SLEs represent a flexible educational system that leverages emerging pedagogical approaches and technologies to enhance student learning experiences. SLE theories align with Gardner’s ideas by advocating for environments that adapt to the needs of individual learners, thus supporting a more personalized and effective educational experience. Integrating these theories into educational practices will foster innovative learning environments that are responsive to students’ diverse needs [22]**.**

**Table 1:** The alignment of Gardner’s multiple intelligence theory domains with the core competencies of public health professionals

Theory of multiple intelligence domains	Public health core competencies	Examples of activities that show alignment
Bodily-kinesthetic intelligence	Cultural competency and diversity skills	Applying hands-on techniques and cultural sensitivity in health interventions
Existential intelligence	Cultural competency and diversity skills	Addressing existential questions in the context of cultural diversity and health equity
Interpersonal intelligence	Leadership and systems thinking skills	Collaborating with diverse stakeholders to lead public health initiatives
Intrapersonal intelligence	Cultural Competency and Diversity Skills	Reflecting on personal values to implement culturally appropriate public health strategies
Linguistic intelligence	Communication skills	Effectively conveying public health information through verbal and written communication
Logical-mathematical intelligence	Analytic and assessment skills	Utilizing logical reasoning and analytical skills to assess public health data and trends
Musical intelligence	Communication skills	Incorporating music and creative arts in health communication and education efforts
Naturalist intelligence	Policy development and planning skills	Identifying and addressing public health issues related to the natural environment
Visual-spatial intelligence	Policy development and program planning skills	Utilizing visual representations for health program planning and assessment

Gardner’s and SLE theories are a natural fit for designing and implementing SLEs. SLEs are systems that innovatively use pedagogical approaches and emerging technologies to support and enhance student learning experiences [22]. SLEs foster active engagement in online settings, reinforcing knowledge and promoting the application of new concepts. SLEs can provide diverse and adaptive learning pathways that cater to the individual strengths and preferences of learners. For example, students with logical-mathematical intelligence might engage with content through problem-solving tasks, whereas those with linguistic intelligence might benefit from text-based resources and discussions. This alignment ensures that learners are engaged and can maximize their potential by interacting with the content in ways that resonate with their unique cognitive profiles.

Furthermore, SLEs incorporate elements of self-regulated learning (SRL) theory, which emphasizes the importance of students taking responsibility for their learning by setting goals, monitoring their progress, and adapting strategies as needed [49]. SLEs can foster SRL through active engagement, creating a positive and supportive learning environment that encourages students to apply new concepts effectively. SLEs use technology to deliver content and encourage SRL by actively supporting learners to enhance their engagement and outcomes [30].

Despite the shift toward technology-enhanced learning, many students may still be accustomed to traditional classroom settings and may need to develop the skills required for success in online learning environments. By aligning with Gardner’s and SLE theories, SLEs can address these challenges and create opportunities for a more inclusive and adaptable educational experience [48].

## Enhancing Smart Learning Environments

SLEs should meet certain conditions to be effective. First, clear instructions should be provided to reduce ambiguity and allow students to understand and engage with the material more effectively [24, 36]. Providing students with additional support and resources, such as multimedia materials, interactive simulations, or online tutoring, can help them overcome content difficulties. These resources provide additional learning and practice opportunities, alternative explanations, and personalized assistance [1]. Regular and timely constructive feedback from educators assists students in identifying areas for growth, correcting misconceptions, and deepening their understanding [24].

In contrast, several negative factors can potentially hamper students’ experiences with content [35]. Inadequate communication and interaction between educators and students can impede comprehension. Students’ progress can also be hampered by a lack of clarification, delayed responses to inquiries, and limited opportunities for discussion [24, 29]. Imposing short deadlines for completing online assignments can cause time constraints and hinder students’ progress. A lack of opportunities for active learning, such as collaborative projects, discussion blogs, or interactive self-directed activities, can hamper students’ understanding and application of content. A lack of interaction and engagement with peers and a lack of experiential learning opportunities can contribute to difficulties in navigating SLEs [47]. SLEs should thus focus on the flow of information to avoid the above pitfalls.

## High-Level Assessment in Online Learning

When designed using Bloom’s taxonomy, high-level assessments in online learning can significantly enhance student engagement and motivation [4]. By incorporating these cognitive levels into assessments, educators can design tasks that challenge students to move beyond memorization to higher-order thinking skills such as analysis, evaluation, and creation. Such assessments encourage deeper learning, foster critical thinking, and make learning more interactive, increasing students’ engagement and motivation as they feel more invested in their educational journey (Table 2). Critical thinking and creativity-based assessments can pique students’ interest, promote deeper understanding, and foster a sense of ownership over their learning [27]. Evaluation, analysis, and synthesis assessments can improve knowledge of the subject matter, develop critical thinking skills, and improve the application of knowledge in real-world situations [5, 6].

**Table 2:** Teaching and learning opportunities aligned with the theory of multiple intelligences in public health education with proposed online activities

Theory of multiple intelligence in public health education	Teaching and learning opportunities in an online environment	Online activities
Applying hands-on techniques and cultural sensitivity in health interventions	Conduct online role-plays and simulations to practice culturally competent healthcare delivery	Online role-playing exercises and cultural competency simulations
Addressing existential questions in the context of cultural diversity and health equity	Engage students in online debates and discussions on ethical and equity issues in public health	Online debate and discussion forums on ethical public health dilemmas
Collaborating with diverse stakeholders to lead public health initiatives	Assign group projects and online team-based activities to develop leadership skills	Online group projects and collaborative problem-solving tasks
Reflecting on personal values to implement culturally appropriate public health strategies	Encourage self-reflection through online journals and individual learning portfolios	Online journaling and self-assessment activities
Effectively conveying public health information through verbal and written communication	Facilitate online discussions, group chats, and written assignments to practice communication skills	Online group discussions and online debates on public health topics
Utilizing logical reasoning and analytical skills to assess public health data and trends	Engage students in data analysis exercises using online data visualization tools and interactive simulations	Data analysis challenges and quizzes using online analytical tools
Incorporating music and creative arts in health communication and education efforts	Explore health-related songs, podcasts, and creative projects as part of health education	Creating health-related podcasts, music videos, or creative art pieces
Identifying and addressing public health issues related to the natural environment	Organize online case studies and debates on environmental health policies and their impact	Online field trips to explore environmental health challenges
Utilizing visual representations for health program planning and assessment	Incorporate visual aids, infographics, and mind maps into online presentations and lectures	Designing and creating online infographics and visual presentations

Online learning can promote learning at higher levels of Bloom’s taxonomy [4]. Active learning opportunities, such as case-based discussions, problem-solving tasks, and collaborative projects, are common in online learning environments (Table 2). These activities encourage students to think critically and analyze, and synthesize information [32]. SLEs can leverage a wide range of multimedia resources, interactive simulations, and real-world applications, allowing students to explore complex topics, apply knowledge to authentic situations, and engage in higher-order thinking [9, 18]. Reflective activities, such as online journals, blogs, or discussion boards, are frequently included in SLEs and encourage students to analyze their learning experiences, connect concepts, and develop critical thinking skills [40, 42]. Social learning theories focus on collaborative, interactive knowledge co-construction [19, 39]. Tools like discussion boards, video conferencing, simulations, and gamification enhance engagement and meaningful learning [2, 7, 9, 31, 38].

## Integrated Framework for Creating Smart Learning Environments

Garrison, Anderson, and Archer [14] developed the Community of Inquiry (CoI) framework, which provides a comprehensive model for understanding and facilitating online learning experiences [14]. The framework comprises three main components: social presence, cognitive presence, and teaching presence. It emphasizes developing a supportive online community, engaging students in meaningful cognitive activities, and ensuring effective instructional design. This paper proposes a conceptual framework that integrates SLEs, SRL, and the CoI in the context of rapidly evolving educational technologies and pedagogical practices.

**Fig. 1:**
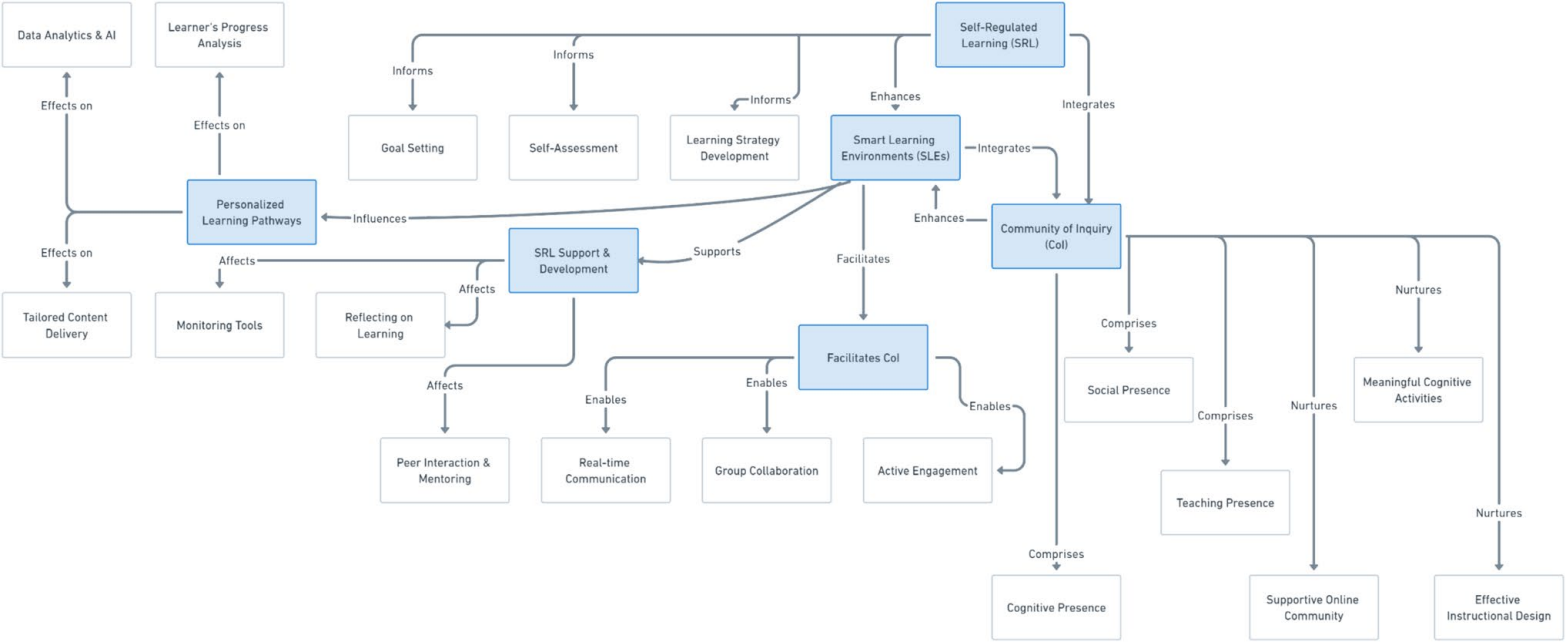
Conceptual framework of the community of inquiry (CoI) framework for smart learning environments

In the proposed conceptual framework (Fig. 1), the SLE acts as a facilitator, seamlessly incorporating both SRL and the CoI framework to enhance the learning experience. The SLE offers students personalized learning pathways on the basis of self-assessment and goal-setting within a supportive learning community.

### Personalized Learning Pathways

An SLE can leverage data analytics and artificial intelligence algorithms to analyze students’ progress and adapt content delivery. SLEs provide tailored content and activities to support individual needs where students can set learning goals and preferences.

### SRL Support and Development

Within an SLE, students can be guided to become self-regulated students. Students can monitor their progress, reflect on their learning process, and develop effective learning strategies. SLEs also encourage peer interaction and peer mentoring to support SRL.

### Collaborative Learning Within CoIs

SLEs facilitate the creation of a robust CoI by promoting real-time communication, group collaboration, and active engagement. Students can participate in online discussions, group projects, and peer reviews, fostering social and cognitive presence within the community.

## Conclusions

Advancing teaching in online learning environments necessitates a multifaceted approach that integrates cutting-edge technologies with pedagogical frameworks tailored to diverse learner needs. The proposed conceptual framework, which combines SLEs, SRL, and the CoI framework, offers a comprehensive strategy for enhancing student engagement, promoting deeper learning, and fostering a supportive online community. By leveraging adaptive technologies and personalized learning pathways, educators can create more inclusive and dynamic educational experiences that cater to individual strengths and preferences. Additionally, fostering a strong sense of social and cognitive presence in online courses can mitigate the challenges of isolation and disengagement, thus enhancing overall learning outcomes. Anonymity in online environments, while sometimes empowering, can also lead to disengagement and reduced accountability. Designing for identity, visibility, and authentic interaction may help counter these effects—an often overlooked but important aspect of online learning that merits further attention.

## Knowledge Gaps and Future Research

Despite the potential of SLEs, there are still gaps in understanding the long-term impacts of these environments on learner outcomes, particularly in public health education. Future research should explore the effectiveness of SLEs in diverse cultural and socioeconomic contexts, the scalability of personalized learning in large online courses, and the role of artificial intelligence in monitoring and supporting student well-being. Additionally, the best practices for integrating SRL into SLEs should be investigated. Similarly, the impact of SLEs on fostering critical thinking and problem-solving skills should be clarified. To advance teaching and learning in online environments, academic institutions must develop comprehensive training programs that equip educators with the skills to effectively utilize SLEs and incorporate SRL strategies. Additionally, academic institutions should invest in adaptive learning technologies that provide personalized learning pathways and real-time feedback. Moreover, online courses should be designed so that they foster community building through collaborative learning, peer interaction, and engagement, enhancing students’ sense of belonging and motivation in the digital learning space.

## Data Availability

The datasets used and/or analyzed during the current study are presented within the article. However, further information is available from the corresponding author on reasonable request.

## Abbreviations

CoI: Community of inquiry
SLE: Smart learning environment
SRL: Self-regulated learning
